# Extremity Ultrasound vs. Computed Tomography at the Third Lumbar Vertebra Level for Assessing the Subcutaneous Adipose Tissue-to-Muscle Ratio

**DOI:** 10.3390/nu18060988

**Published:** 2026-03-20

**Authors:** Arabella Fischer-Hammerschmied, Maximilian Pesta, Anatol Hertwig, Timo Siebenrock, Ricarda Hahn, Martin Anwar, Konstantin Liebau, Isabel Timmermann, Jonas Brugger, Martin Posch, Helmut Ringl, Dietmar Tamandl, Cecilia Veraar, Andrea Lassnigg, Martin Bernardi, Edda Tschernko, Joop Jonckheer, Martin Sundström Rehal, Michael Hiesmayr

**Affiliations:** 1 Division of Cardiothoracic and Vascular Anaesthesia and Intensive Care Medicine, Department of Anaesthesia, Intensive Care and Pain Medicine, Medical University of Vienna, 1090 Vienna, Austria; maximilian.pesta@meduniwien.ac.at (M.P.); andrea.lassnigg@meduniwien.ac.at (A.L.); edda.tschernko@meduniwien.ac.at (E.T.); 2 Center for Medical Statistics, Informatics and Intelligent Systems, Medical University of Vienna, 1090 Vienna, Austria; 3 Department of Biomedical Imaging and Image-Guided Therapy, Medical University of Vienna, 1090 Vienna, Austria; 4 Department of Intensive Care, Universitair Ziekenhuis Brussel, 1090 Brussels, Belgium; 5 Department of Perioperative Medicine and Intensive Care (PMI), Karolinska University Hospital Huddinge, 141 86 Stockholm, Sweden; martin.sundstrom.rehal@ki.se; 6 Division of Anaesthesia and Intensive Care, Department of Clinical Science, Intervention and Technology (CLINTEC), Karolinska Institute, 171 77 Stockholm, Sweden

**Keywords:** adipose tissue, muscles, ultrasonography, computed tomography, X-ray, body composition

## Abstract

**Background/Objectives:** A ratio of subcutaneous adipose tissue to muscle mass may be more informative than defining low subcutaneous adipose tissue and muscle mass separately. The objective of this study was to determine which ultrasound measurement points in the upper and lower extremities predict the subcutaneous adipose tissue (SAT)-to-muscle ratio as measured by gold-standard computed tomography (CT) at the third lumbar vertebra (L3) level. **Methods:** Two hundred hospitalised patients (41% female; median (Q1–Q3) age: 61.3 (51.0–70.1) years) who underwent an abdominal CT scan for any clinical reason within 48 h prior to extremity ultrasound were included in this prospective observational study conducted from 2017 to 2019. Ultrasound measurements of subcutaneous adipose tissue and muscle thickness were obtained at three measuring points on the thigh and two on the upper arm. On the CT scan at the L3 level, subcutaneous (SAT) and visceral adipose tissue and skeletal muscle area were measured. A linear LASSO (Least Absolute Shrinkage and Selection Operator) model was used to identify which ultrasound sites best predicted the CT L3 SAT-to-muscle ratio. **Results:** Height, weight, sex, SAT-to-muscle ratio at four ultrasound measuring points and abdominal circumference predicted the CT SAT-to-muscle ratio in the LASSO model (R^2^ = 0.70; cross-validated R^2^ = 0.63; *p* values are not reported in LASSO regression and R^2^ is used instead). The upper-arm anterolateral ultrasound site most strongly influenced the CT SAT-to-muscle ratio (estimate × standard deviation of predictor: 0.24). **Conclusions:** The CT SAT-to-muscle ratio at the L3 level can be predicted non-invasively using bedside ultrasound, particularly at the anterolateral measuring point of the upper arm. Bedside ultrasound assessment of the ratio of subcutaneous adipose tissue to muscle on the anterolateral upper arm provides a within-patient comparison of body compartments.

## 1. Introduction

Total skeletal muscle area at the third lumbar vertebra (L3) on CT is a widely used proxy for whole-body muscle mass [1]. Low muscle mass is central to the diagnosis of malnutrition [2] and has been associated with longer hospital stays [3] and higher mortality [4]. However, defining low muscle mass using fixed cut-offs is problematic. Published cut-offs differ in how they adjust for body stature, whether they use age- or BMI-specific subgroups, and how thresholds are derived statistically [5]. As a result, the estimated prevalence of low muscle mass can range widely, from 5 to 86%, depending on the chosen cut-off [5].

Rather than using height- or weight-adjusted cut-offs to compare muscle mass between patients, we propose a practical approach that compares body compartments within the same patient. A ratio of subcutaneous adipose tissue (SAT) to muscle may be more informative than defining low SAT and muscle mass separately. This ratio indicates whether a patient has relatively less, the same, or more SAT than muscle; this can give an estimate of overall body composition. An imbalance between SAT and muscle mass is often observed, with athletes typically exhibiting relatively low levels of adipose tissue relative to muscle, whereas sarcopenic obesity is associated with relatively high adipose tissue levels. Furthermore, the relationship between adipose tissue and muscle mass may provide valuable prognostic information, such as risk of death and postoperative complications [6,7,8,9].

Furthermore, ultrasound can be used to quantify the SAT-to-muscle ratio without the need for patient transport or the use of radiation. It remains to be demonstrated whether the SAT-to-muscle ratio can be measured using ultrasound on the easily accessible upper and lower extremities, and which regions best correlate with the SAT-to-muscle ratio assessed by the gold-standard CT at the L3 level. This translational step—from bedside extremity ultrasound to the prediction of the CT L3 SAT-to-muscle ratio—is a key novelty of the present study and clinically relevant when CT is unavailable for serial assessment.

This prospective study aimed to determine whether bedside ultrasound can provide a valid estimate of the CT SAT-to-muscle ratio at the L3 level in hospitalised patients, as well as which upper- and lower-extremity measuring points can be used to achieve this.

## 2. Materials and Methods

### 2.1. Study Design and Population

This prospective observational study (clinicaltrials.gov identifier: NCT03160222) was performed at the Medical University of Vienna from 2017 to 2019. Surgical or medical inpatients who had undergone an abdominal CT scan for any clinical reason in the last 48 h were screened in the hospital digital management system. Patients were enrolled consecutively on days when a trained study team member was available (AFH, MP, AH, TS, RH, MA, KL, IT). CT eligibility and selection criteria are described below (see Section 2.2). Patients younger than 18 years were excluded. After informed consent was obtained, the study-related ultrasound examination was performed. Ethical approval was obtained from the Ethics Committee of the Medical University of Vienna, and the study was conducted in accordance with the Declaration of Helsinki. Reporting followed the STROBE guidelines for observational studies [10]. We targeted a sample size of 200 to allow a linear model with up to 10 predictors, consistent with the convention of at least 10 observations per predictor [11], and to evaluate model performance using cross-validation.

### 2.2. Computed Tomography

We selected a single axial CT scan at the level of the third lumbar vertebra L3 with both transverse processes visible. Scans were contrast-enhanced in the portovenous phase with a slice thickness of 3 mm. Tube current and peak kilovoltage were variable. Muscle, SAT and visceral adipose tissue (VAT) areas were measured using Hounsfield unit thresholds of −29 to 150 HU for muscle [12], −190 to −30 HU for SAT [13,14] and −150 to −50 HU for VAT [14,15]. All CT examinations were clinically indicated and therefore used field-of-view settings tailored to the organs of interest. When the lateral SAT was partially outside the field of view, the missing contour was manually retraced (Supporting Information, Figure S3). In some scans, a dorsal or lateral fascial structure with positive HU was present within the SAT compartment; this structure was excluded from adipose tissue segmentation, as confirmed by two radiologists (DT, HR) (Supporting Information, Figure S4). If kidneys were visible on the CT scan, any perirenal or hilar adipose tissue was additionally marked semi-automatically. CT SAT-to-muscle and VAT-to-muscle area ratios were computed. The CT measurements were performed semi-automatically in OsiriX by 6 examiners (AFH, MPe, MA, AH, RH, IT) and verified by consensus between two radiologists (DT, HR). Pseudonymised CT scans were analysed after patient recruitment was completed.

### 2.3. Ultrasound Examination

An illustrated guide to the ultrasound examination and the results of its reliability assessment have been published in detail [16,17]. Briefly, 2 measuring points at 70% of the upper arm length and 3 measuring points at 50% of the thigh length were marked on each side of the body (Figure S1, Supporting Information) [16]. Upper arm and thigh lengths were determined according to specific anatomical landmarks [16]. At each measuring point, a scan in the short- and long-axis was performed with minimal compression using a gel pad and gel. Minimal compression was verified via the blurred borders of the scan. Subcutaneous adipose tissue thickness was measured from the skin surface, including the dermis, to the adipose–muscle interface, starting at the centre of the scan along the imagined shortest line to the bone surface. We included the muscle fascia in the subcutaneous adipose tissue thickness, since it is easier to delimit the muscle fascia from muscle tissue than from subcutaneous adipose tissue [16]. Muscle thickness was measured from below the muscle fascia to the bone surface. The ultrasound (US) SAT-to-muscle thickness ratio was then computed. In this study, eight examiners performed the ultrasound examinations. Prior to the study, one experienced examiner (AFH) trained the other novice examiners (MPe, AH, TS, RH, MA, KL, IT). The intra- and inter-examiner reliability of the ultrasound examination was previously published [17].

### 2.4. Clinical Examination

Abdominal circumference was measured at the level of the umbilicus, which lies at the level of the third lumbar vertebra [18]. Additionally, the presence of oedema was assessed in upper and lower extremities. The Functional Comorbidity Index (FCI) was also assessed [19].

### 2.5. Statistical Analysis

Continuous variables were described as median (Q1–Q3). Differences in continuous variables between sexes were assessed with Mann–Whitney U-tests. In descriptive analyses, the means of all ultrasound values of both body sides were computed. Pearson correlations between ultrasound SAT and CT SAT and VAT were described.

Missing abdominal circumference values were imputed by CT circumference in 35 patients since abdominal and CT circumference were highly correlated (R^2^ = 0.86). Both-handers were randomly classified as right- or left-handers. Ultrasound measurements were performed twice at all measuring points in 120 patients to determine intra- or inter-examiner reliability [17]. In these 120 patients, only the first ultrasound run was taken into account in the present analysis.

For the following analyses, the ultrasound SAT-to-muscle ratio on the right side of the body was considered, as 90% of patients were right-handed and measuring only one body side is less time-consuming in clinical practice [12].

Correlations between US ratios at all measuring points on the right side in both planes were visualised in a scatterplot. Correlations between the predictors and the outcome (CT SAT-to-muscle ratio) were visualised in another scatterplot. 

To predict the CT SAT-to-muscle ratio, we imputed missing ultrasound values via the K-Nearest Neighbours (KNN) method. Each missing entry was replaced with the weighted average of that variable from the k = 10 most similar observations in the feature space. The following predictors were considered: ultrasound ratio (SAT thickness/muscle thickness) at the 5 measuring points of the right side, sex, weight, height, age, FCI, admission type (medical vs. surgical), clinical presence of oedema and abdominal circumference. A LASSO (Least Absolute Shrinkage and Selection Operator) regression model was fitted in order to obtain a parsimonious prediction model and mitigate the risk of overfitting [20]. In simple terms, LASSO attempts to find the simplest model with the fewest predictors and smallest coefficients that still predicts well. The data-driven selection and shrinkage in LASSO breaks the usual assumptions behind classical inference. Thus, both standard 95% confidence intervals and *p* values are not valid in LASSO regression and are therefore not reported. R^2^ remains valid in LASSO regression. Two Lasso models were built, both including the predictors described above. The first included ultrasound measuring points in the short-axis plane, while the second included those in the long-axis plane, to avoid collinearity between ultrasound measurements. The penalty or tuning parameter λ in LASSO was determined by 10-fold cross-validation using the one-standard-error rule, because this gives the simplest and most stable LASSO model. Optimism in the R^2^ of the final LASSO prediction model was calculated by 10-fold cross-validation of the entire model fitting procedure. The resulting optimism-adjusted R^2^ was reported.

To assess the relative influence of the selected predictors in the LASSO model, we calculated the change in predicted CT SAT-to-muscle ratio on the right side of the body associated with a one–standard deviation (SD) increase in each predictor (SD × regression estimate).

In a sensitivity analysis, only the highest contributing predictors were included in an ordinary linear regression. For this ordinary linear regression, no imputation for missing data was performed.

A two-sided significance level of 0.05 was applied for all hypothesis tests. As this is an exploratory study, no adjustment of *p* values for multiplicity was performed. R version 4.3.3 or higher and the glmnet package version 4.1-8 were used for statistical analysis.

## 3. Results

### 3.1. Study Population, Ultrasound and CT Scans

Of 1729 screened patients, 200 met the inclusion criteria (Supporting Information, Figure S2) [12]. Detailed baseline characteristics were previously published [12]. Briefly, 41% of participants were female, the median (IQR) age was 61.3 (51.0–70.1) years and the mean BMI was 24.9 ± 4.8 kg·m^−2^. Two thirds were surgical admissions, and 44% had a malignant disease. Of the 4000 planned ultrasound scans (5 measuring points × 2 planes × 2 body sides × 200 patients), 98% could be performed and evaluated. The remaining 2% were missing because the muscle fascia or bone surface could not be identified or because scanning was not feasible due to upper-arm or thigh bandages [12]. On CT, HU-based segmentation incompletely captured oedematous SAT in 3 scans and oedematous VAT in 12 scans (Supporting Information, Figure S5). In 29 scans, lateral SAT was partially outside the field of view, so the missing contour was manually retraced (Supporting Information, Figure S3).

### 3.2. Description of SAT-to-Muscle Ratio in Ultrasound and CT

**Figure 1:**
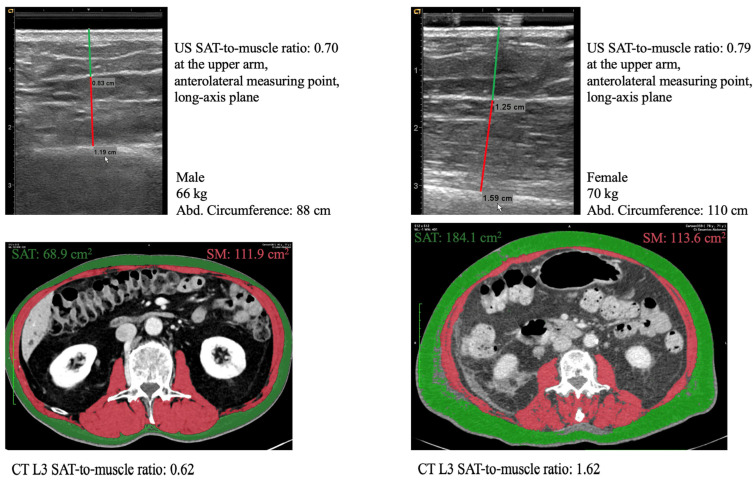
Ultrasound and CT SAT-to-muscle ratio in two representative patients. SAT: subcutaneous adipose tissue in green. SM: skeletal muscle in red. Please see Figure S6 for both the short- and long-axis planes to help distinguish SAT from muscle in the ultrasound scans.

All ultrasound- and CT-derived SAT values were significantly higher in women than in men (*p* < 0.001) (Table 1). The muscle values from ultrasound and CT assessments have been published previously [12]. Ultrasound and CT SAT-to-muscle ratios from two representative patients are shown in Figure 1 and Figure S6.

Across all ultrasound measurement sites, women had higher SAT-to-muscle ratios than men in both the upper and lower extremities (*p* < 0.001). The ultrasound SAT-to-muscle ratio also differed by anatomical site. For instance, in women, the median SAT-to-muscle ratio was 0.71 (0.60–0.84) at the ventral thigh site and increased to 1.06 (0.69–1.43) at the anterolateral upper-arm site (Table 2). The anterolateral upper-arm site showed the highest median ratios in both sexes: 0.57 (0.42–0.80) in men and 1.06 (0.69–1.43) in women (Table 2).

On CT, women had a higher SAT-to-muscle area ratio than men. The medians were 1.51 (1.01–2.20) and 0.86 (0.59–1.28), respectively (Table 2). The median VAT-to-muscle area ratio was 0.53 (0.15–1.09) in women and 0.96 (0.44–1.54) in men (Table 2).

Mean ultrasound SAT thickness averaged across all measuring points highly correlated with CT SAT area (R^2^ = 0.62, *p* < 0.001). In contrast, lower-extremity ultrasound SAT thickness was not associated with CT VAT (R^2^ = 0.009; *p* = 0.21), and upper-extremity ultrasound SAT thickness showed only a weak association with CT VAT (R^2^ = 0.09; *p* < 0.001). CT SAT was moderately correlated with CT VAT (R^2^ = 0.25; *p* < 0.001).

### 3.3. Prediction of CT SAT-to-Muscle Ratio

Short- and long-axis ultrasound measurements were strongly correlated (Figure S7). Height, weight, all US SAT-to-muscle ratios and abdominal circumference had a visible linear relationship with CT SAT-to-muscle ratio (Figure S8). The variables selected by LASSO (Table 3) corresponded to those displaying the strongest linear relationships in the previous scatterplot (Figure S8). Model fitting was similar for the long- and short-axis measuring points (R^2^ = 0.70 and R^2^ = 0.67, respectively) (Table 3). The final long-axis LASSO model achieved an R^2^ of 0.70 (Table 4), with model and residual plots shown in Figures S9 and S10. Optimism was 0.07, corresponding to a cross-validated R^2^ of 0.63 after optimism correction.

The largest contributions to the predicted CT ratio came from the anterolateral ultrasound SAT-to-muscle ratio, followed by abdominal circumference, weight and sex, each showing substantial changes in the predicted CT ratio per 1-SD increase (Table 5).

In sensitivity analyses, an ordinary linear regression model including only sex, weight and abdominal circumference yielded an R^2^ of 0.60, which improved to 0.68 when including the US ratio at the anterolateral measuring point (Supporting Information, Tables S1 and S2).

## 4. Discussion

The CT SAT-to-muscle ratio at L3 can be predicted non-invasively using bedside ultrasound. The ultrasound SAT-to-muscle ratio at the anterolateral measuring point of the upper arm contributed most to predicting the CT SAT-to-muscle ratio.

### 4.1. Prediction of CT SAT-to-Muscle Ratio

The anterolateral upper-arm ultrasound SAT-to-muscle ratio contributed most to the prediction of the CT SAT-to-muscle ratio. Compared to SAT of the thigh, SAT of the upper arm may better reflect abdominal SAT, which could explain its stronger predictive value. Upper extremity and trunk SAT can promote metabolic syndrome [21,22,23]. In contrast, lower-extremity SAT is often considered metabolically protective, showing slower free fatty acid turnover, greater adipocyte hyperplasia and lower inflammatory activity [23].

Weight, sex and abdominal circumference were also important predictors and explained a substantial proportion of the variability in the CT ratio. Nevertheless, including the anterolateral upper-arm ultrasound ratio was warranted because it provided the largest contribution and improved model performance. This model improvement is central to clinical applicability, because it suggests that a brief bedside ultrasound can refine assessment of body composition when CT is unavailable or impractical. In line with this, ultrasound-derived adipose measures have been reported to correlate with MRI-based visceral-to-subcutaneous adipose distribution more strongly than anthropometric measures such as weight, BMI or waist or hip circumferences [24].

Short- and long-axis ultrasound measurements exhibited similar prediction performance. Because both planes can be obtained reliably at all sites [17], either plane can be used for assessment. When tissue interfaces are difficult to delineate, such as in oedema, acquiring both planes may facilitate identification of the muscle fascia separating SAT from muscle [12,16].

### 4.2. Potential Clinical Value of the SAT-to-Muscle Ratio

Our study demonstrated that bedside ultrasound can provide a valid estimate of the CT SAT-to-muscle ratio. The clinical relevance of the SAT-to-muscle ratio depends on three dimensions: bedside feasibility, practicability and the potential value for diagnosis, prognosis, or treatment planning. Regarding bedside feasibility, weight and sex are routinely available from clinical records, abdominal circumference is easy to obtain with a measuring tape and upper-arm ultrasound is quick (approximately 5 min), non-invasive and radiation-free. Regarding practicability, the SAT-to-muscle ratio is methodologically simple. Cut-offs for low muscle mass vary widely by adjustment strategy (height, weight, BMI), subgrouping and statistical derivation [5], producing large differences in estimated prevalence [5]. The SAT-to-muscle ratio provides a practical overview of body composition within the same patient without requiring adjustment for body stature. Regarding potential clinical value, prior studies suggest that combined adipose–muscle metrics are more consistently associated with outcomes such as mortality and postoperative complications than adipose or muscle measurements alone [6,7,8,9]. Evidence regarding adipose tissue area or muscle area alone is less consistent, with some studies identifying adipose tissue as the key correlate [6,7,9] and others emphasising muscle [25,26,27,28]. By integrating both compartments, the SAT-to-muscle ratio captures the relative balance between energy stores and contractile tissue and may therefore offer a more stable and clinically meaningful predictor of outcomes than SAT or muscle alone.

In summary, bedside ultrasound estimation of the SAT-to-muscle ratio may simplify body composition assessment and enhance outcome prediction when CT is unavailable. It may also allow longitudinal monitoring of body composition to guide nutrition, medication dosing and rehabilitation decisions, particularly in patients experiencing prolonged hospital stays or with complex medical histories.

### 4.3. SAT and VAT Are Two Distinct Regional Entities

Some authors suggest grouping upper-body SAT with VAT, as both have been linked to increased metabolic risk [23]. High VAT levels are associated with insulin resistance and systemic inflammation [23,29]. However, in our study, upper-extremity or abdominal SAT correlated only very weakly with VAT. Therefore, we challenge the oversimplistic approach of grouping upper-extremity SAT and VAT. Rather, SAT and VAT are two distinct entities, while SAT exhibits different metabolic functions when located in the lower or upper regions.

### 4.4. Strengths and Limitations

The strengths of this study include its prospective design, large sample size, the inclusion of both surgical and medical patients and a stepwise methodological approach that established the ultrasound protocol [16], confirmed intra- and inter-examiner reliability [17] and then validated ultrasound against CT.

One limitation of the work was oedema, which can impair HU-based adipose tissue segmentation. Oedematous SAT and VAT were incompletely captured in 3 and 12 out of 200 CT scans, respectively. As shown previously, ultrasound is similarly affected by oedema [12,16]. We addressed this issue by clinically assessing oedema and offering it as a candidate predictor; however, it was not retained by LASSO under the selected penalty, suggesting limited incremental predictive value in this setting. Another limitation was the partial truncation of lateral SAT in 29 of 200 CT scans. When lateral SAT was partially outside the field of view, the missing contour was manually retraced (Supporting Information, Figure S3). The study was conducted at a single centre, which may limit generalisability.

## 5. Conclusions

Bedside extremity ultrasound can predict the SAT-to-muscle ratio measured by gold-standard CT at the L3 level. The anterolateral upper-arm ultrasound SAT-to-muscle ratio provided the strongest predictive contribution. This bedside approach offers a practical within-patient comparison of adipose tissue and muscle compartments.

## Figures and Tables

**Table 1:** Ultrasound SAT thickness and CT L3 SAT and VAT areas (n = 200 patients).

		All (n = 200)		Male (n = 118)		Female (n = 82)		*p*
Ultrasound SAT thickness at measuring points (cm), median (Q1–Q3)								
Thigh	ventral s-a	1.11	(0.84–1.58)	0.93	(0.75–1.14)	1.54	(1.19–1.93)	<0.001
	ventral l-a	1.15	(0.87–1.57)	0.98	(0.78–1.19)	1.52	(1.21–1.97)	<0.001
	lateral s-a	1.19	(0.78–1.75)	0.83	(0.67–1.21)	1.69	(1.35–2.21)	<0.001
	lateral l-a	1.18	(0.81–1.85)	0.85	(0.68–1.18)	1.72	(1.30–2.29)	<0.001
	medial s-a	1.61	(1.21–2.18)	1.37	(1.10–1.76)	2.10	(1.61–2.56)	<0.001
	medial l-a	1.66	(1.23–2.23)	1.37	(1.11–1.78)	2.19	(1.66–2.43)	<0.001
Upper arm	anterior s-a	0.58	(0.49–0.78)	0.56	(0.44–0.70)	0.64	(0.53–0.92)	<0.001
	anterior l-a	0.61	(0.50–0.81)	0.57	(0.44–0.73)	0.70	(0.57–0.95)	<0.001
	anterolateral s-a	1.01	(0.76–1.40)	0.91	(0.67–1.14)	1.26	(0.95–1.72)	<0.001
	anterolateral l-a	1.04	(0.79–1.46)	0.92	(0.66–1.13)	1.32	(0.93–1.77)	<0.001
CT measurements (cm^2^), median (Q1–Q3)								
	L3 SAT area	142.2	(92.3–211.8)	134.2	(83.1–196.6)	156.6	(107.6–242.9)	0.02
	L3 VAT area	92.0	(31.3–185.6)	136.4	(63.4–231.0)	57.6	(15.7–118.0)	<0.001

s-a: short-axis plane, l-a: long-axis plane, SAT: subcutaneous adipose tissue, VAT: visceral adipose tissue. Ultrasound SAT thickness values were averaged across the right and left sides of the body. *p* values for differences between men and women are presented.

**Table 2:** Ultrasound ratios and CT L3 ratios (n = 200 patients).

		All (n = 200)		Male (n = 118)		Female (n = 82)		*p*
Ultrasound SAT-to-muscle ratio (SAT thickness/muscle thickness) at measuring points (cm), median (Q1–Q3)								
Thigh	ventral s-a	0.52	(0.34–0.71)	0.38	(0.29–0.52)	0.71	(0.60–0.84)	<0.001
	ventral l-a	0.50	(0.34–0.73)	0.38	(0.30–0.51)	0.72	(0.60–0.87)	<0.001
	lateral s-a	0.38	(0.22–0.55)	0.23	(0.19–0.38)	0.55	(0.42–0.70)	<0.001
	lateral l-a	0.36	(0.22–0.56)	0.24	(0.19–0.37)	0.56	(0.45–0.68)	<0.001
	medial s-a	0.63	(0.41–0.86)	0.48	(0.36–0.66)	0.77	(0.65–0.99)	<0.001
	medial l-a	0.58	(0.41–0.82)	0.47	(0.35–0.64)	0.77	(0.62–0.99)	<0.001
Upper arm	anterior s-a	0.24	(0.19–0.31)	0.20	(0.16–0.25)	0.29	(0.25–0.42)	<0.001
	anterior l-a	0.25	(0.20–0.36)	0.21	(0.16–0.26)	0.34	(0.26–0.46)	<0.001
	anterolateral s-a	0.70	(0.45–1.02)	0.54	(0.41–0.78)	0.98	(0.68–1.33)	<0.001
	anterolateral l-a	0.71	(0.47–1.09)	0.57	(0.42–0.80)	1.06	(0.69–1.43)	<0.001
CT L3 ratios, median (Q1–Q3) [min–max]								
	SAT-to-muscle ratio (SAT area/SM area)	1.08	(0.65–1.67) [0.01–4.71]	0.86	(0.59–1.28) [0.01–2.98]	1.51	(1.01–2.20) [0.14–4.71]	<0.001
	VAT-to-muscle ratio (VAT area/SM area)	0.75	(0.30–1.41)	0.96	(0.44–1.54)	0.53	(0.15–1.09)	<0.001

s-a: short-axis plane, l-a: long-axis plane, SAT: subcutaneous adipose tissue, VAT: visceral adipose tissue, SM: skeletal muscle. Ultrasound SAT thickness values were averaged across the right and left sides of the body. *p* values for differences between men and women are presented.

**Table 3:** LASSO model selection to predict the CT SAT-to-muscle ratio.

Models Including Measuring Points in the	Independent Variables	R^2^
short-axis plane	weight, height, sex, abdominal circumference, lateral, medial, anterior, anterolateral	0.67
long-axis plane	weight, height, sex, abdominal circumference, lateral, medial, anterior, anterolateral	0.70

Models including measuring points in the short- or long-axis plane on the right side of the body to predict CT SAT-to-muscle ratio. The possible independent variables for Lasso model selection included the ultrasound SAT-to-muscle ratio at the 5 measuring points in the short-axis plane, sex, weight, height, age, FCI (Functional Comorbidity Index), admission type (medical vs. surgical), oedema and abdominal circumference.

**Table 4:** Final model for predicting CT SAT to muscle ratio (n = 200), R^2^ = 0.70.

	Estimate of CT SAT-to-Muscle Ratio
Weight (kg)	0.0101
Height (cm)	−0.0061
Male sex = 1, Female sex = 0	−0.3193
Abdominal circumference (cm)	0.0118
US SAT-to-muscle ratio at lateral l-a measuring point of thigh	0.2138
US SAT-to-muscle ratio at medial l-a measuring point of thigh	0.3184
US SAT-to-muscle ratio at anterior l-a measuring point of upper arm	0.2015
US SAT-to-muscle ratio at anterolateral l-a measuring point of upper arm	0.4859

Final prediction model: CT SAT-to-muscle ratio = −0.1495 + (0.0101 × weight) − (0.0061 × height) − (0.3193 × sex) + (0.0118 × abdominal circumference) + (0.2138 × US SAT-to-muscle ratio at lateral l-a measuring point of right thigh) + (0.3184 × US SAT-to-muscle ratio at medial l-a measuring point of right thigh) + (0.2015 × US SAT-to-muscle ratio at anterior l-a measuring point of right upper arm) + (0.4859 × US SAT-to-muscle ratio at anterolateral l-a measuring point of right upper arm), where male sex = 1, female sex = 0 (reference), weight (kg), height (cm), ultrasound SAT-to-muscle ratio (no unit). The range of weight in our study population was 41 to 118 kg. The range of height in our study population was 148 to 197 cm. The range of abdominal circumference was 65 to 145 cm. The range of US ratio on the right side was 0.09 to 2.06 at the lateral l-a point, 0.10 to 2.38 at the medial l-a point, 0.07 to 1.84 at the anterior l-a point and 0.10 to 3.17 at the anterolateral l-a point. For example, using a male-to-female proportion of 0.59, an average patient—with a weight of 74 kg, a height of 172 cm, an abdominal circumference of 98 cm and ultrasound ratios of 0.43, 0.64, 0.29 and 0.80 at the selected measuring points—had a predicted CT SAT-to-muscle ratio of 1.26. SAT: subcutaneous adipose tissue, l-a: long-axis, US: ultrasound.

**Table 5:** Contribution of each independent variable for predicting the CT ratio.

Predictor	Mean	SD	Change in Predicted CT Ratio for a One-SD Increase in the Predictor (=SD × Estimates #)
Weight (kg)	74	16	0.16
Height (cm)	172	9	−0.05
Male = 1, Female = 0	0.59 *	0.49	−0.16
Abd. Circ. (cm)	98	15	0.18
US ratio lateral l-a	0.43	0.27	0.06
US ratio medial l-a	0.64	0.34	0.11
US ratio anterior l-a	0.29	0.19	0.04
US ratio anterolateral l-a	0.80	0.49	0.24

l-a: long-axis. The indicated US ratios are on the right side of the body. # The estimates of each predictor are presented in Table 4. * as proportion of men to women.

## Data Availability

The data presented in this study are available on request from the corresponding author due to privacy restrictions.

## Supplementary Materials

The following supporting information can be downloaded at: https://www.mdpi.com/article/10.3390/nu18060988/s1, Table S1. Supplementary sensitivity analysis for predicting CT SAT–to–muscle ratio from sex, weight and abdominal circumference; Table S2. Supplementary sensitivity analysis for predicting CT SAT–to–muscle ratio from sex, weight, abdominal circumference and ultrasound SAT–to–muscle ratio at the anterolateral measuring point of the upper arm; Figure S1. Ultrasound measuring points; Figure S2. Flow chart; Figure S3. Example of a CT scan, where (A) lateral borders of SAT are slightly cut off and where (B) lateral borders of SAT area were manually retraced; Figure S4. Example of a CT scan, where a fascial structure with positive HU was seen in the SAT area.; Figure S5. Examples of oedematous CT scans, where (A) subcutaneous or (B) visceral adipose tissue was not entirely marked within HU boundaries; Figure S6. Ultrasound SAT–to–muscle ratio in the short- and long-axis plane in two exemplary patients; Figure S7. Scatterplot with correlations between US ratios at all measuring points in both planes (as separate file); Figure S8. Scatterplot with correlations between all variables (as separate file); Figure S9. Final model plot: visual correlation between predicted and real CT Ratio; Figure S10. Residual plot of the final model.

## Abbreviations

The following abbreviations are used in this manuscript:

CT	computed tomography
FCI	Functional Comorbidity Index
KNN	K-Nearest Neighbours
LASSO	Least Absolute Shrinkage and Selection Operator
L3	third lumbar vertebra
SAT	subcutaneous adipose tissue
SM	skeletal muscle
SMA	skeletal muscle area
US	ultrasound
VAT	visceral adipose tissue
