# Supplementary material for: Extremity Ultrasound vs. Computed Tomography at the Third Lumbar Vertebra Level for Assessing the Subcutaneous Adipose Tissue-to-Muscle Ratio
Source: Nutrients. 2026 Mar 20;18(6):988. doi: 10.3390/nu18060988 (PMC13029642; doi:10.3390/nu18060988)
Supplement: Supplementary file 1 [file nutrients-18-00988-s001.zip › nutrients-4106177-supplementary.pdf]

Online Supporting Information

Extremity ultrasound vs. computed tomography at the third lumbar vertebra level for assessing the subcutaneous adipose tissue-to-muscle ratio

Arabella Fischer-Hammerschmied, MD PhD[1*]; Maximilian Pesta, MD[1]; Anatol Hertwig, MD[1]; Timo Siebenrock, MD[1]; Ricarda Hahn[1]; Martin Anwar, MD[1]; Konstantin Liebau, MD[1]; Isabel Timmermann, MD[1]; Jonas Brugger[2]; Martin Posch, Dr. Prof.[2]; Helmut Ringl, MD Prof.[3]; Dietmar Tamandl MD Prof.[3]; Cecilia Veraar, MD PhD[1]; Andrea Lassnigg, MD Prof[1]; Martin Bernardi, MD Prof[1]; Edda Tschernko, MD Prof[1]; Joop Jonckheer MD Prof[4]; Martin Sundström Rehal, MD PhD[5,6]; Michael Hiesmayr, MD Prof.[2]

[1]Division of Cardiothoracic and Vascular Anaesthesia and Intensive Care Medicine, Department of Anaesthesia, Intensive Care and Pain Medicine, Medical University of Vienna; [2]Center for Medical Statistics, Informatics and Intelligent Systems, Medical University of Vienna; [3]Department of Biomedical Imaging and Image-guided Therapy, Medical University of Vienna; [4]Department of Intensive Care, Universitair ziekenhuis Brussel, Brussels, Belgium; [5]Department of Perioperative Medicine and Intensive Care (PMI), Karolinska University Hospital Huddinge, Stockholm, Sweden; [6]Division of Anaesthesia and Intensive Care, Department of Clinical Science, Intervention and Technology (CLINTEC), Karolinska Institute, Stockholm, Sweden
*Corresponding Author: arabella.fischer-hammerschmied@meduniwien.ac.at

Table of contents

Tables

Table S1. Supplementary sensitivity analysis for predicting CT SAT–to–muscle ratio from sex, weight and abdominal circumference

Table S2. Supplementary sensitivity analysis for predicting CT SAT–to–muscle ratio from sex, weight, abdominal circumference and ultrasound SAT–to–muscle ratio at the anterolateral measuring point of the upper arm

Figures

Figure S1. Ultrasound measuring points

Figure S2. Flow chart

Figure S3. Example of a CT scan, where (A) lateral borders of SAT are slightly cut off and where (B) lateral borders of SAT area were manually retraced

Figure S4. Example of a CT scan, where a fascial structure with positive HU was seen in the SAT area.

Figure S5. Examples of oedematous CT scans, where (A) subcutaneous or (B) visceral adipose tissue was not entirely marked within HU boundaries

Figure S6. Ultrasound SAT–to–muscle ratio in the short- and long-axis plane in two exemplary patients

Figure S7. Scatterplot with correlations between US ratios at all measuring points in both planes (as separate file)

Figure S8. Scatterplot with correlations between all variables (as separate file)

Figure S9. Final model plot: visual correlation between predicted and real CT Ratio

Figure S10. Residual plot of the final model

Figures were created in R version 4.3.3 (or higher), PowerPoint (version 16.78) or Word (version 16.78).

	Estimate (95% CI) of CT SAT–to–muscle ratio
Male sex	-1.109563 (-1.277338034 to -0.94178815)
Weight (kg)	0.018046 (0.009152509 to 0.02693887)
Abdominal Circumference (cm)	0.020821 (0.011729861 to 0.02991256)

Table S1. Supplementary sensitivity analysis for predicting CT SAT–to–muscle ratio from sex, weight and abdominal circumference (n=200), $R^2 = 0.60$, $P < 0.001$

CT SAT–to–muscle ratio = -1.452520 - (1.109563 x male sex) + (0.018046 x weight) + (0.020821 x abdominal circumference)
where male sex=1, female sex=0, weight (kg), abdominal circumference (cm)

	Estimate (95% CI) of CT SAT–to–muscle ratio
Male sex	-0.784742 (-0.959292517 to -0.61019202)
Weight (kg)	0.015486 (0.007511134 to 0.02346122)
Abdominal Circumference (cm)	0.015708 (0.007484733 to 0.02393158)
Ultrasound SAT–to–muscle ratio at anterolateral l-a measuring point of upper arm	0.608672 (0.444290918 to 0.77305290)

Table S2. Supplementary sensitivity analysis for predicting CT SAT–to–muscle ratio from sex, weight, abdominal circumference and ultrasound SAT–to–muscle ratio at the anterolateral measuring point of the upper arm (n=198), $R^2 = 0.68$, $P < 0.001$

CT SAT to muscle ratio = -1.449187 - (0.784742 x male sex) + (0.015486 x weight) + (0.015708 x abdominal circumference) + (0.608672 x ultrasound SAT–to–muscle ratio at anterolateral l-a measuring point of upper arm)
where male sex=1, weight (kg), height (cm), ultrasound SAT–to–muscle ratio (no unit), l-a: long-axis plane

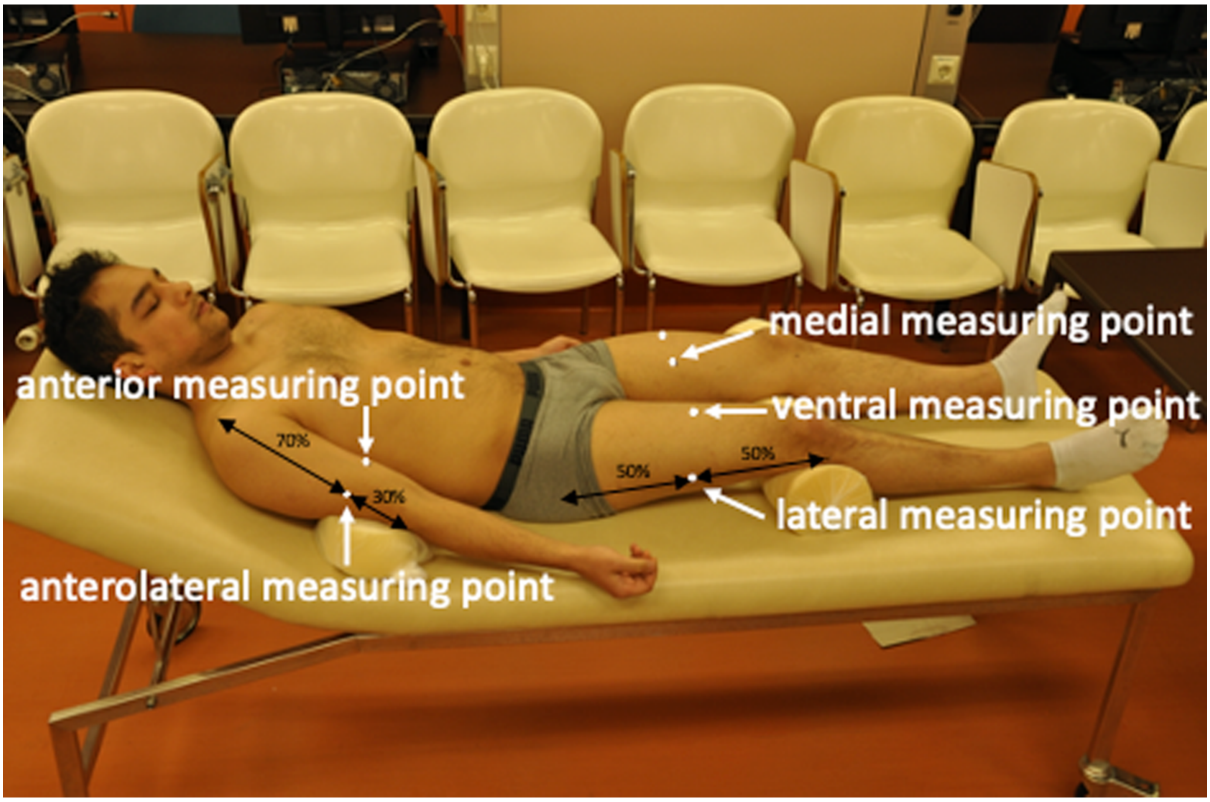

Figure S1. Ultrasound measuring points

Reprinted and adapted from Clinical Nutrition Experimental, 32:38–73, Fischer A, Anwar M, Hertwig A, Hahn R, Pesta M, Timmermann I, et al. Ultrasound method of the USVALID study to measure subcutaneous adipose tissue and muscle thickness on the thigh and upper arm: an illustrated step-by-step guide 2020, licensed under CC BY-NC-ND 4.0 (https://creativecommons.org/licenses/by-nc-nd/4.0/)

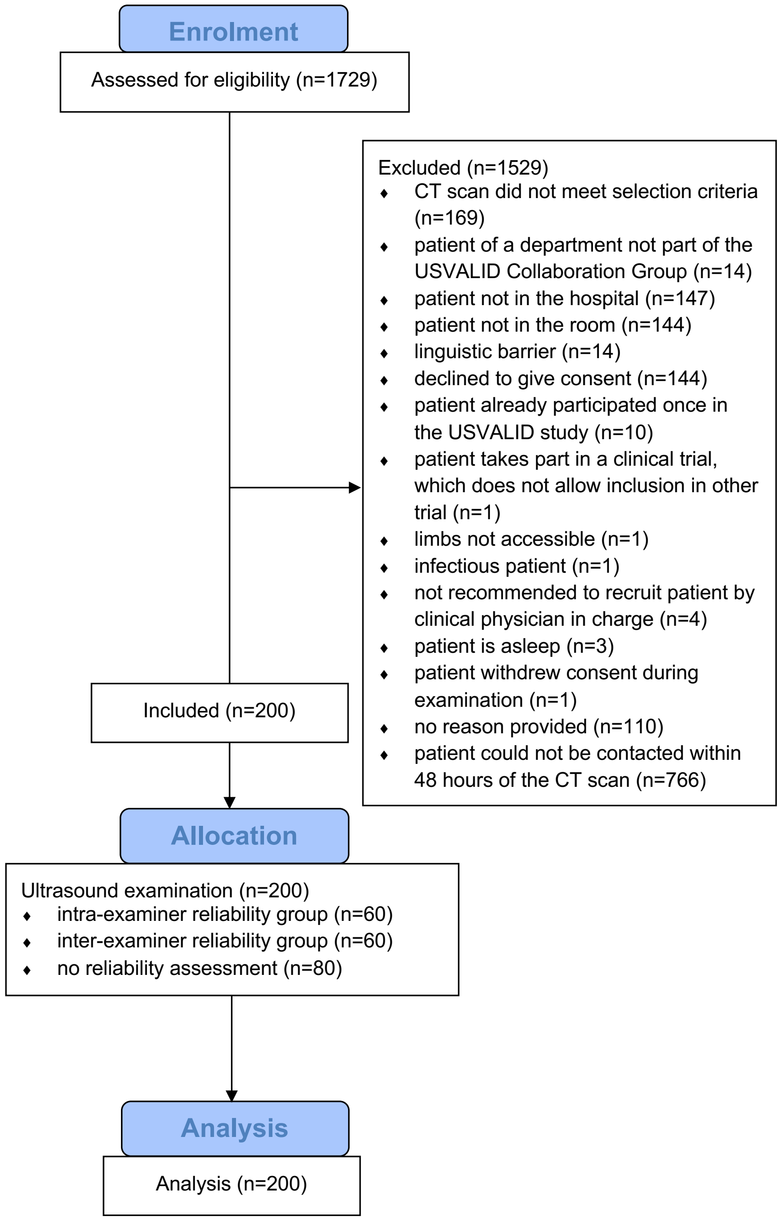

Figure S2. CONSORT flow chart.

Reprinted from Clinical Nutrition, Fischer A, Hertwig A, Hahn R, Anwar M, Siebenrock T, Pesta M, et al. Validation of bedside ultrasound to predict lumbar muscle area in the computed tomography in 200 non-critically ill patients: The USVALID prospective study, Copyright (2022), with permission from Elsevier

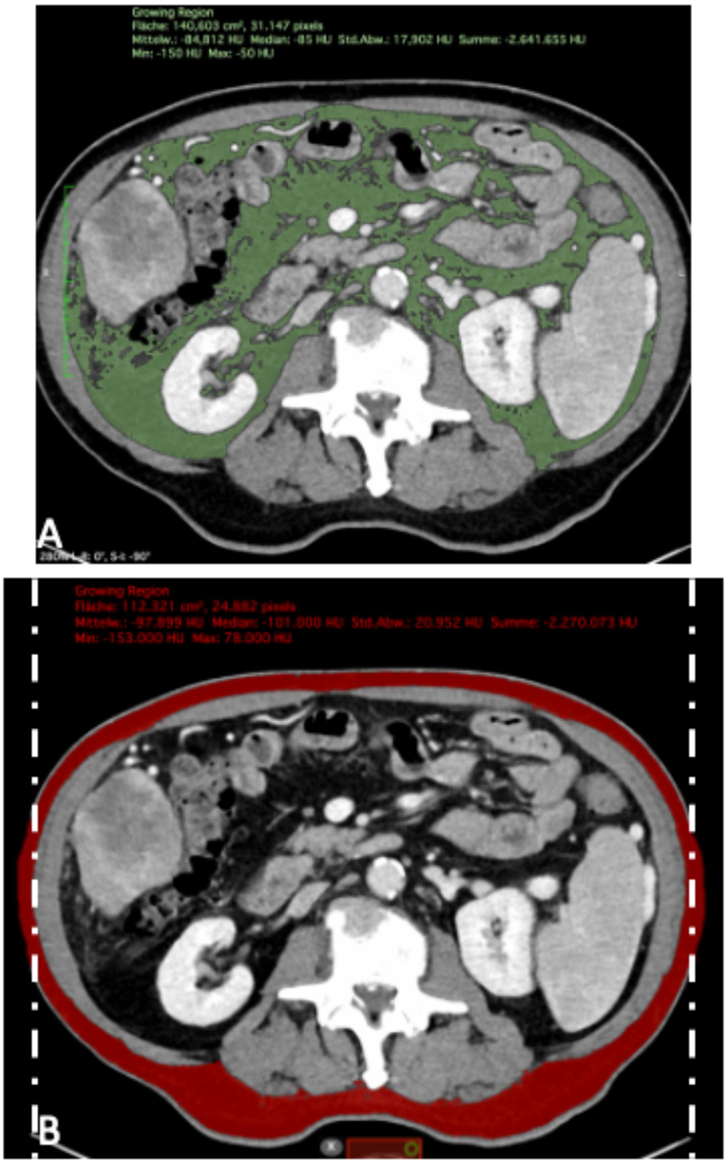

Figure S3. Example of a CT scan, where (A) lateral borders of SAT are slightly cut off and where (B) lateral borders of SAT area were manually retraced

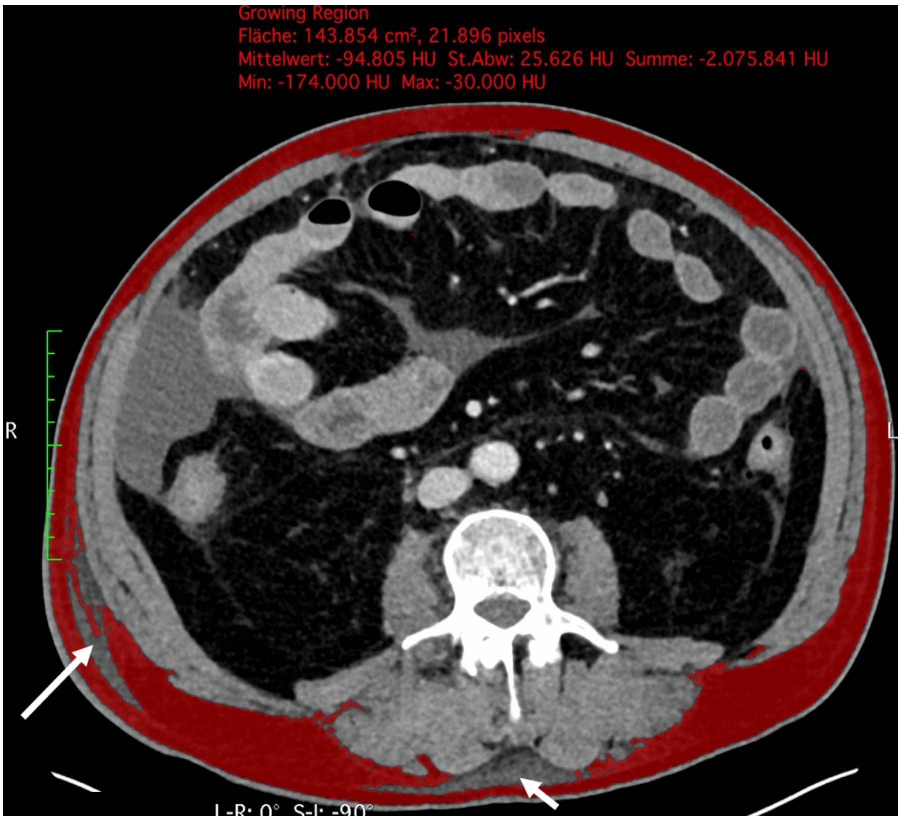

Figure S4. Example of a CT scan, where a fascial structure with positive HU was seen in the SAT area.

The fascial structure is not part of adipose tissue.

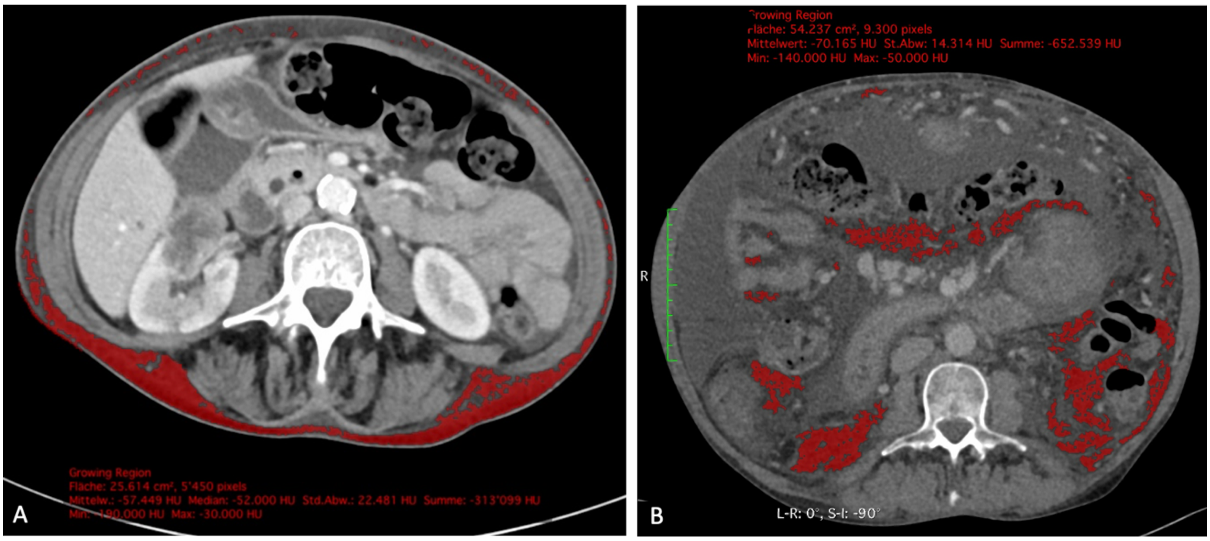

Figure S5. Examples of oedematous CT scans, where (A) subcutaneous or (B) visceral adipose tissue was not entirely marked within HU boundaries

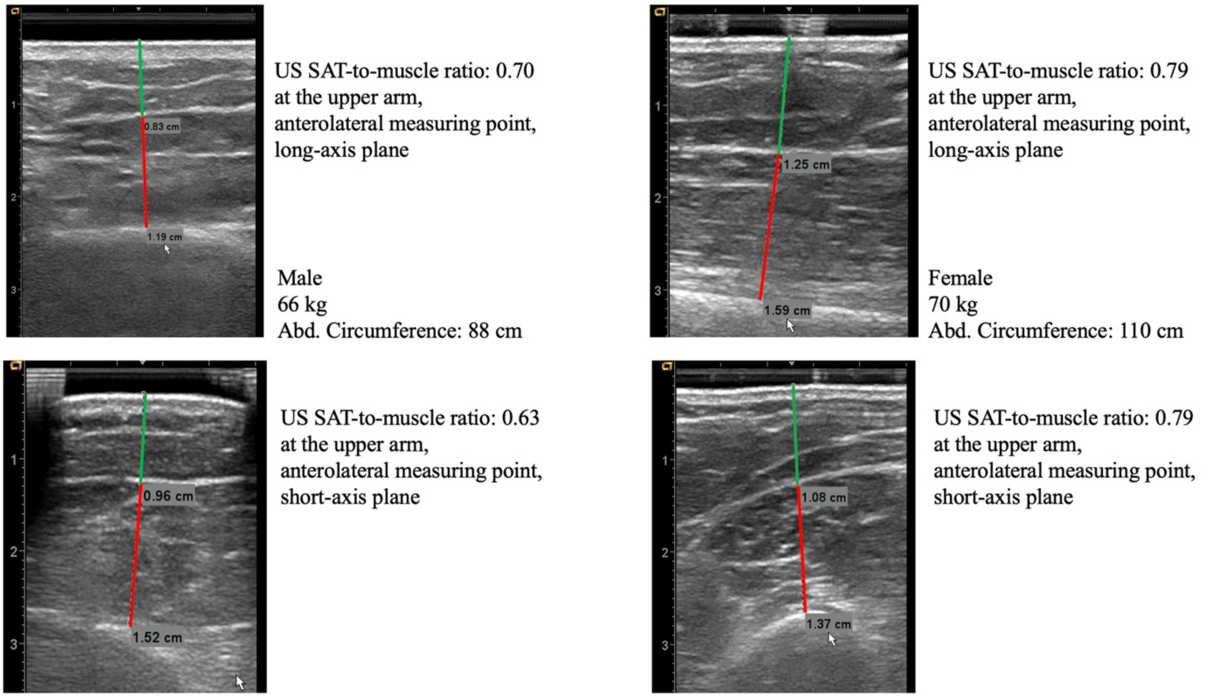

Figure S6. Ultrasound SAT–to–muscle ratio in the short- and long-axis plane in two exemplary patients

Figure S7 (See separate file). Scatterplot with correlations between US ratios at all measuring points in both planes on the right side

Figure S8 (See separate file). Scatterplot with correlations between all variables

Only US ratios on the right side in the long-axis are presented here, since US ratios highly correlate between the long- and short-axis plane (Figure S7).

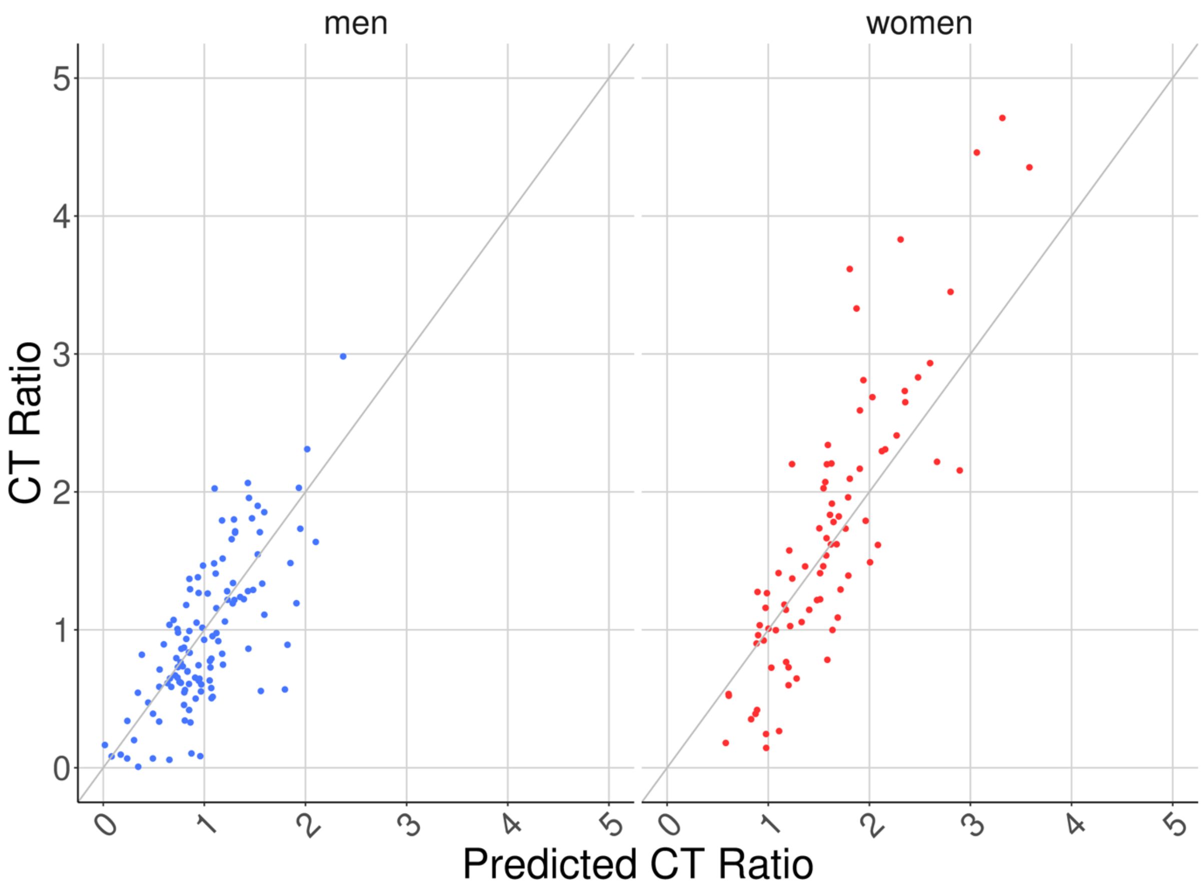

Figure S9. Final model plot: visual correlation between predicted CT Ratio by final model (Table 4) and real CT Ratio, n=200

The diagonal line represents the main diagonal or line of equality (x=y).

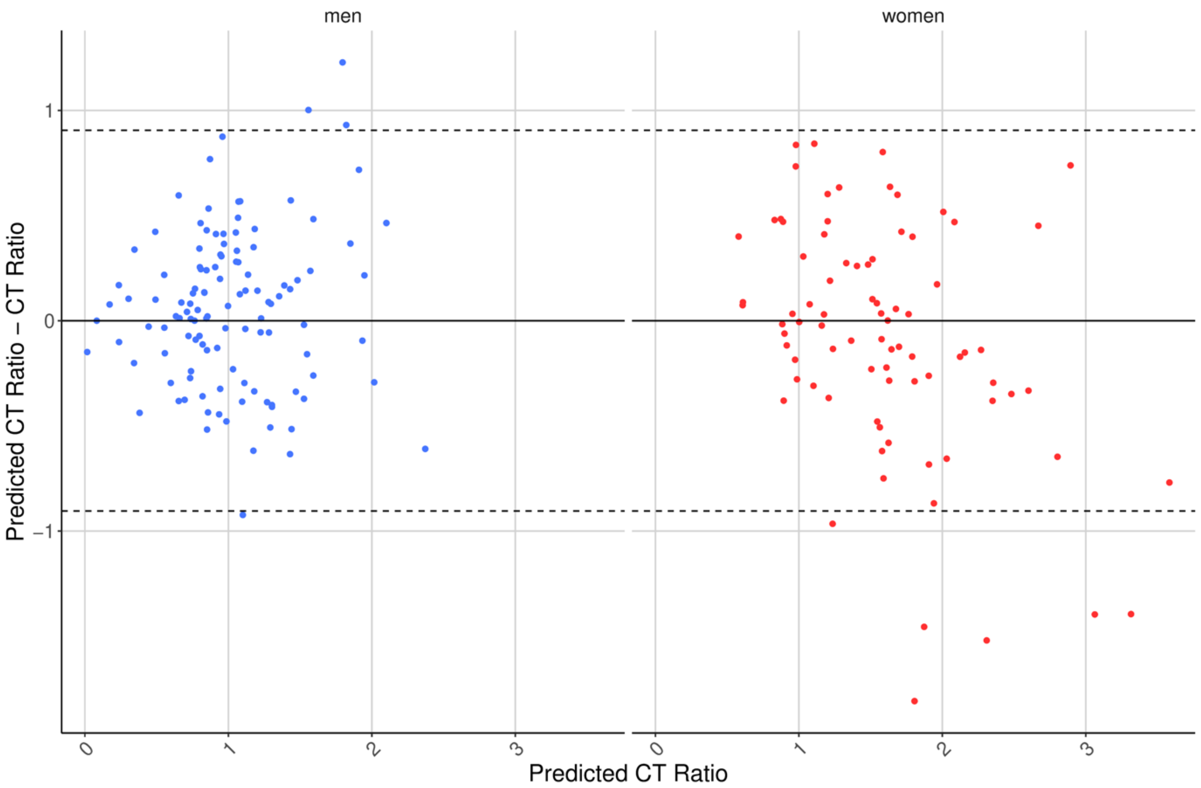

Figure S10. Residual plot of the final model, n=200

The solid line represents the mean difference of 0 (95% CI, -0.06 to 0.06). The dashed lines represent the 95% limits of agreement (mean - (1.96 x SD), mean + (1.96 x SD)) of -0.91 and 0.91.
